# Anti-inflammatory and antiproliferative activities of date palm pollen (*Phoenix dactylifera*) on experimentally-induced atypical prostatic hyperplasia in rats

**DOI:** 10.1186/1476-9255-8-40

**Published:** 2011-12-23

**Authors:** Ahmed A Elberry, Shagufta T Mufti, Jaudah A Al-Maghrabi, Essam A Abdel-Sattar, Osama M Ashour, Salah A Ghareib, Hisham A Mosli

**Affiliations:** 1 Department of Clinical Pharmacy, Faculty of Pharmacy, King Abdulaziz University, Jeddah, Saudi Arabia; 2 Department of Pathology, Faculty of Medicine, King Abdulaziz University, Jeddah, Saudi Arabia; 3 Department of Pharmacognosy, Faculty of Pharmacy, Cairo University, Cairo, Egypt; 4 Department of Pharmacology and Toxicology, Faculty of Pharmacy, King Abdulaziz University, Jeddah, Saudi Arabia; 5 Department of Urology, Faculty of Medicine, King Abdulaziz University, Jeddah, Saudi Arabia

**Keywords:** atypical prostatic hyperplasia, date palm pollen, anti-inflammatory, antiproliferative, cytokines, immunohistochemistry, castration, citral

## Abstract

**Background:**

Atypical prostatic hyperplasia (APH) is a pseudoneoplastic lesion that can mimic prostate adenocarcinoma because of its cytologic and architectural features. Suspension of date palm pollen (DPP) is an herbal mixture that is widely used in folk medicine for male infertility. The aim of the present study was to evaluate the effect of DPP suspension and extract on APH-induced rats.

**Methods:**

APH was induced in adult castrated Wistar rats by both s.c. injection of testosterone (0.5 mg/rat/day) and smearing citral on shaved skin once every 3 days for 30 days. Saw palmetto (100mg/kg), DPP suspension (250, 500 and 1000 mg/kg), and lyophilized DPP extract (150,300 and 600 mg/kg) were given orally daily for 30 days. All medications were started 7 days after castration and along with testosterone and citral.

**Results:**

The histopathological feature in APH-induced prostate rats showed evidence of hyperplasia and inflammation. Immunohistochemical examination revealed that the expressions of IL-6, IL-8, TNF-α, IGF-1 and clusterin were increased, while the expression of TGF-β1 was decreased that correlates with presence of inflammation. Moreover, histopathological examination revealed increased cellular proliferation and reduced apoptosis in ventral prostate. Both saw palmetto and DPP treatment has ameliorated these histopathological and immunohistochemical changes in APH-induced rats. These improvements were not associated with reduction in the prostatic weight that may be attributed to the persistence of edema.

**Conclusion:**

DPP may have a potential protective effect in APH-induced Wistar rats through modulation of cytokine expression and/or upregulation of their autocrine/paracrine receptors.

## Introduction

Benign prostatic hyperplasia (BPH) is a hormone- and age-related disease, characterized by histological changes and variable increases in prostatic size [1]. Atypical prostatic hyperplasia (APH) or adenosis is a pseudoneoplastic lesion that can mimic prostate adenocarcinoma because of its cytologic and architectural features. APH is usually an incidental finding in transurethral resections or simple prostatectomies performed in the clinical setting of BPH [2]. In these prostate diseases, there is an imbalance between prostate cell growth and apoptosis. This imbalance is complex and influenced by factors that stimulate proliferation and minimize cell apoptosis such as growth factors, cytokines and steroid hormones [3].

The role of inflammation in prostate diseases is suggested by the presence of inflammatory cells within the prostate in BPH patients [4]. The more the inflammation, the larger the prostate will be [5]. Inflammation is a complex phenomenon consisting of a humoral (cytokines) and cellular (leukocytes, monocytes and macrophages) components [3]. Inflammation is usually a self-limited event, with initial pro-inflammatory cytokines, growth factor release and angiogenesis followed by anti-inflammatory cytokine-mediated resolution [6]. Chronic inflammation continuously produces inducible cyclooxygenase (COX-2) that increases the production of prostaglandin-E2 (PGE2) and reduces the E-c adherin protein [7]. Chronic inflammation also produces free radicals as various reactive oxygen species (ROS) [8].

Suspension of *Phoenix dactylifera *date palm pollen (DPP) is an herbal mixture that is widely used as a folk remedy for curing male infertility in traditional medicine [9]. A thousand tonnes of DPP are produced every year by millions of palm trees grown in the Arabian region. DPP differed from bee pollen in that it is of a known source and its homogeneity, purity and is easily to be standardized. DPP was reported to have gonadal stimulating potency [10], as well as fertility promotion in women in ancient Egypt [11]. It was reported that date pollen grain extracts contain estrogenic materials, estrone, as gonad-stimulating compounds that improve male infertility and exhibit gonadotrophin activity in the rat [11,12]. Cernilton is another pollen extract derived from several different plants in southern Sweden and has been known to be effective in the treatment of chronic abacterial prostatitis and prostatodynia [13,14].

The aim of this study was to investigate the protective effect of DPP suspension and its alcoholic extract on the histpathological changes related to inflammation, proliferation and/or apoptosis in APH using citral and testosterone-induced APH model in castrated rats.

## Methods

### Chemicals and reagents

Antibodies against clusterin, phospho-Smad2, and β-actin were obtained from Santa Cruz Biotechnology (Santa Cruz, CA; anti-clusterin for Western blot analysis); Upstate Biotechnology [Lake Placid, NY; anti-clusterin for immunohistochemistry (IHC)]; and Cell Signaling Technology Inc. (Danvers, MA). Antibody against TGF-β1 ligands were purchased from Dakocytomation (Carpinteria, CA). Citral was obtained from Fluka Chemie AG, Buchs, Switzerland. Testosterone was obtained from Sigma-Aldrich and Dakocytomation (Carpinteria, CA).

### Collection and extraction of date pollen sample

Date palm pollen sample was collected from El-Katawiah, El-Sharkia, Egypt. It was collected in March 2010 and kept in 20°C till extraction. 1250 g DPP powder was extracted with 80% ethanol (3 × 5 liters) by using Ultraturrax T25 homogeniser (Janke and Kunkel, IKA Labortechnik, Stauten, Germany) at a temperature not exceeding 25°C. The extract was evaporated under reduced pressure, lyophilized to give 240 g of yellowish semisolid residue and protected from light at 4°C until use.

### Experimental animals

Adolescent male Wistar rats, aged 50-60 days, were obtained from the animal facility of King Fahd Research Center, King Abdulaziz University, Jeddah, Saudi Arabia. They were used in the study according to the guidelines of the Biochemical and Research Ethics Committee at King Abdulaziz University, in accordance with the NIH guidelines. Animals were housed in a well-ventilated, temperature-controlled room at 22 ± 3°C with a 12 h light-dark cycle. They were provided with standard rat chow pellets and tap water. All experimental procedures were performed between 8-10 a.m. and care was taken to avoid stressful conditions.

Orchidectomy was performed aseptically, under ethyl ether anesthesia, by a midscrotal incision. Following ligation of the spermatic cord and vessels, testes and epididymides were removed. The remaining stump was pushed back through the inguinal canal into the abdominal cavity, and the scrotal sac was closed by sutures [15]. After castration, the rats were maintained under standard laboratory conditions for 7 days in order to allow a definite involution of the prostatic gland [16].

APH was induced as previously described by Engelstein et al. [17] in castrated rats using citral and testosterone. Rats were subcutaneously injected with testosterone propionate in corn oil (0.5 mg/0.1 ml/rat) each day for 30 days. Citral, 1 M diluted in 70% ethanol, was smeared on a different shaved area of skin, each time, on the back at a final dose of 185 mg/kg every 4^th ^day for 30 days. Control groups were smeared with the solvent (ethanol) alone at the shaved skin. Considering the pungent lime fragrance of citral, the control groups were kept in a separate location from the citral-treated animals to avoid possible false results as citral have some influence via the olfactory tract [17].

### Animal treatment

Seven days after castration, the animals were randomly divided into nine groups (n = 7). Group I served as shamed operation received corn oil (0.1 ml/rat) and 1% CMC-Na (0.3 ml/100 g body wt) daily during the period of the experiment. Groups from II-IX were castrated and had APH. Group II served as negative control and received CMC-Na 1% as previously mentioned. Group III served as positive control and received saw palmetto (100 mg/kg). Groups from IV-VI were treated with DPP suspension (DPPS) at doses of 250, 500 or 1000 mg/kg/day respectively by oral gavage. Groups from VII-IX were treated with 150, 300, or 600 mg/kg/day of DPP extract (DPPE) respectively. All the DPP powder and extract were suspended in 1% CMC-Na and were given to rats once daily by oral gavage along with testosterone injection and citral smearing for 30 days as described before by Scolnik et al. [18]. After scarifaction, ventral prostate of each rat in each group were removed from the body, 24 hr after the last administration, and weighed. Half of the tissues were frozen in liquid nitrogen and stored at -75°C until use. The remaining tissues were fixed immediately in 0.1 M phosphate-buffered 10% formalin (pH 7.4) for 48 h and then embedded in paraffin and were used for histological studies. Serial 4-μm-thick sections from each tissue specimen were prepared and mounted on poly-L-lysinecoated glass slides. These were used for detection of IL-6 and TGF-β1 receptors by immunohistochemistry.

### Histopathology and Histoscore

A part of the ventral lobes were separated and fixed overnight in Stieve's solution. Thereafter, the tissue was thoroughly rinsed with water, and immersed overnight in ethanol 70%. Then, it was dehydrated, embedded in paraffin, and 5 mm thick sections were cut and stained by Harris' hematoxylin eosin, according to standard procedures [19].

A score-chart protocol (histoscore) developed by Scolnik et al. [18] was used to obtain an objective quantitative assessment (Table 1). The examination, description, and scoring of the slides were performed in a blinded manner. The scoring system presented in arbitrary units to make a better evaluation. In a second step, the cumulative score in each group were correlated to the final histological diagnosis in order to establish a range score for normal and hyperplasia. Additional histological inflammatory score described by De Nunzio et al. [20] was used to evaluate the inflammation. Score 0: no inflammation, Score 1: scattered inflammatory cell infiltrate without nodules, score 2: no confluent lymphoid, score 3: Large inflammatory areas with confluence.

**Table 1:** Cumulative chart score of histological findings (Arbitrary Units) in rat ventral prostate [19]

***Strain***	Experimental group	**Animal no**.
		**subtotal**
***Low-Power Magnification***		
***Luminal shape***	regular (1); villous (3); papillary (4); cribriform (5)	------
***Acinar shape***	tubular (1); branched (3); irregular (5)	------
***Interacinar shape***	large or moderate (1); back-to-back glands (5)	------
***Stroma***	fine (1); abundant (3); fibrosis/severe smooth muscle hyperplasia (5)	------
***High-Power Magnification***		
***Epithelial shape**:*	flattened (1); cuboidal (1); cylindrical (3); hexagonal (5)	------
***Number of layers**:*	mono- (1); oligo, 2-4 (3); pluri, > 5 (5) If > 1, then add: focal (3); diffuse (5)	------
***Alignment:***	Polar (1); apolar (3) If there is piling up of epithelial cells add (3) If there is budding out of epithelial cells into stroma add (5) If periacinar clusters of epithelial cells are found add (3) If isolated clusters of epithelial cells are found outside acini add (5)	------
***Lesion distribution***	Unilobar: isolated (2); multiple (6) Bilobar: isolated (4); multiple (8)	------
***Nuclear shape:***	Round, regular (1); irregular (5) Small (2), large (2), small and large in the same acinus (4)	------
***Mitoses per field**:*	absent (0); isolated, 1-2 (2); abundant, 3-5 (5); excessive, > 5 (10)	------
***Basement membrane:***	Intact (1); interrupted (5) Thin (1); thick (5)	------
*Total score (arbitrary units)*		------

*Other Findings: *edema; congestion; hemorrhages; pyknotic nuclei-epithelial/stromal; mononuclear exudate-stromal/intraluminal

### Immunohistochemistry and Immunhistoscoring

Hematoxylin and eosin staining was performed to observe histopathology. For analysis of IL-6 and TGF-β1 expression, sections from the paraffin embedded tissue blocks were mounted on charged glass slides and baked at 60°C for 1 hr in the oven, then mounted in Ventana staining machine. Dewaxed by EZ Prep (Xylene substitute) and rehydrated. The tissue sections were heated in Ventana buffer CC1 (pH 6) to facilitate antigen retrieval, treated with H_2_O_2 _to eliminate endogenous peroxidase. This was followed by incubation for 60 min at room temperature with primary antibodies IL-6 and TGF-β1. The dilutions used were; IL-6 (dilution 1:50) and TGF-β1 (dilution of 1:25). Subsequently, the sections were incubated with biotinylated secondary antibody using avidin-biotin complex method. The immunoreaction was visualized using diaminobenzidine. All sections were lightly counterstained with hematoxylin as a back ground. The positive controls used for IL-6 and TGF-βR1 were from the colon. The negative controls comprised serial sections that were stained using equivalent concentrations of nonimmune mouse IgG in place of the primary antibodies. The level of staining was evaluated independently by three observers blinded to experimental conditions.

Expression of TGF-β1 and IL-6 was evaluated according to a semiquantitative scale: -0, no detectable staining at all; 1, less than 10% of the cells stained positive; 2, 10 -50% positive cells; and 3, more than 50% of cells positive [21]. Staining intensity was scored as 0 (no detectable stain), 1 (weak staining detected at intermediate to high power), 2 (moderate detected at low to intermediate power) to 3 (strong detected at low power) [22].

### Reverse transcriptase-polymerase chain reaction (RT-PCR)

Total RNA was extracted from the snap-frozen tissue samples using total RNA isolation kit (Macherey-Nagel) according to the manufacturer's instructions. RT was performed in a 10 μl reaction mixture. The RT reaction contained 1 μg RNA, 10 mM Tris-HCl (pH 8.3), 50 mM KCl, 1.5 mM MgCl2, 2.5 mM dithiothreitol, 500 μmol/liter each of dATP, dCTP, dGTP, and dTTP, 40 U RNasin, 25 μg/ml oligo d(T)12-18, and 100UMoloney murine leukemia virus reverse transcriptase (Bioline, London, UK). The reaction mixture was incubated at 42°C for 60 min and then heated to 80°C for 5 min. The resultant cDNA was used for PCR. For quantitative real-time RT-PCR, we prepared appropriate dilutions of each single-strand cDNA followed by normalizing of the cDNA content using β-actin as a quantitative control. Quantitative PCR amplification was performed with a 25-μl final volume consisting of 1 μl RT reaction mixture, 3 mM MgCl2, 10 pmol of each sense and antisense primer, and 12.5 μl (Roche Diagnostics) (Table 2). PCR conditions were as follows: initial denaturation at 95°C for 10 min and 35 cycles of denaturation at 94°C for 1 min, annealing at 55°C for 1 min, and elongation at 72°C for 2 sec with a final elongation at 72°C for 10 min. samples were migrated in 1% agarose gel using electrophoresis, UV visualized, and images were analyzed using totallab120 (Nonlinear Dynamic Ltd). Clusterin, TGF-β1, IGF-1, IL-6, IL-8, and TNF-α expression in the test samples were normalized to the corresponding β-actin level and were reported as the relative band intensity to the β-actin gene expression.

**Table 2:** Primer for quantitative reverse transcriptase-polymerase chain reaction (RT-PCR) analysis

Gene	Forward primer	Reverse primer
β-actin	5'-GTCACCCACACTGTGCCCATCT-3'	5'-ACAGAGTACTTGCGCTCAGGAG-3'
IL-6	5'-GAACTCCTTCTCCACAAGCG-3'	5'-TTTTCTGCCAGTGCCTCTTT-3'
IL-8	5'-CTGCGCCAACACAGAAATTA-3'	5'-ATTGCATCTGGCAACCCTAC-3'
TNFα	5'-CAGAGGGAAGAGTTCCCCAG-3'	5'-CCTTGGTCTGGTAGGAGACG-3'
TGF-b	5'-GTTCTTCAATACGTCAGACATTCG-3'	5'-CATTATCTTTGCTGTCACAAGAGC-3'
IGF-1	5'-CACAGGCTATGGCTCCAGCAT-3'	5'-TCTCCAGCCTCCTCAGATCACA-3'
Clusterin	5'-CTGACCCAGCAGTACAACGA-3'	5'-TGACACGAGAGGGGACTTCT-3'

### Statistical analysis

Data were expressed as mean ± SE and were analyzed by analysis of variance (ANOVA) followed by Tukey-Kramer multiple comparisons test. Inflammation scores and their significance were calculated by Chi-square test with Yate's corrections. Differences were considered significant with a *P *value less than 0.05. Statistical analyses were performed using the SPSS for Windows (v. 10.0).

## Results

### Changes in relative prostate weight

Prostatic weights were increased significantly in APH-induced rats. There were no significant changes in relative prostate weight after treatment with either, saw palmetto, DPPS or DPPE (Table 3).

**Table 3:** Body weight (BW), absolute prostate weight (APW), and relative prostate weight (RPW) of rats treated with date palm pollen suspension (DPPS) and the alcoholic extract (DPPE)

Treatment group	BW (gm)		ABW (gm)	RBW (gm)
	start	end		
Normal rats	199.14 ± 5.16	229 ± 8.810	0.106 ± 0.011	0.452 ± 0.051
Rats with APH (negative control)	187.6 ± 8.656	241.4 ± 12.55	0.656 ± 0.056	2.721* ± 0.197
Saw Palmetto (100 mg/kg)	192.8 ± 4.591	258.3 ± 10.22	0.742 ± 0.036	2.919* ± 0.217
DPPS (250 mg/kg)	190 ± 5.024	243.6 ± 11.89	0.752 ± 0.025	3.15* ± 0.215
DPPS (500 mg/kg)	178.3 ± 4.207	221.8 ± 7.127	0.745 ± 0.051	3.38* ± 0.274
DPPS (1000 mg/kg)	193.7 ± 8.929	275 ± 11.547	0.75 ± 0.084	2.79* ± 0.342
DPPE (150 mg/kg)	215 ± 7.693	244.5 ± 8.675	0.719 ± 0.044	2.92* ± 0.193
DPPE (300 mg/kg)	189.7 ± 7.136	232.9 ± 9.425	0.696 ± 0.056	3.039* ± 0.343
DPPE (600 mg/kg)	184 ± 5.219	244.8 ± 9.502	0.752 ± 0.036	3.10* ± 0.197

(n = 7 rats in each group).

*Significantly different from normal rats at p < 0.05.

### Changes in gene expression of IL-6, IL-8 and TNF-α

**Figure 1:**
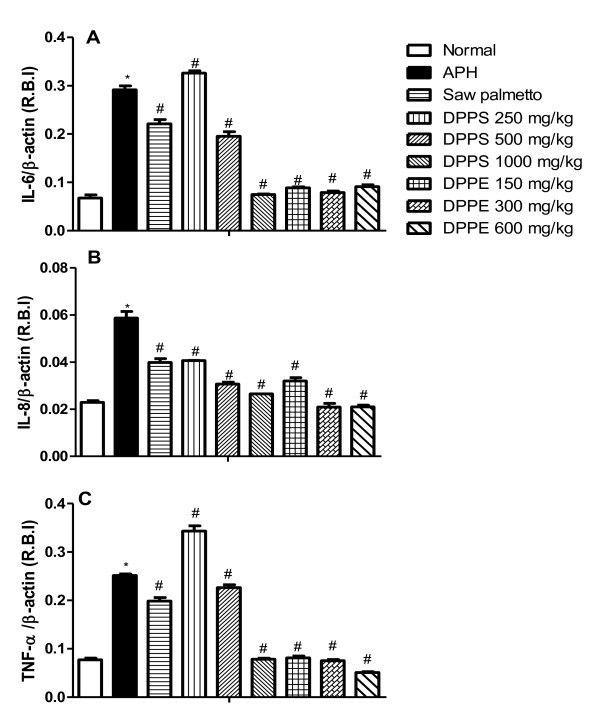
**The expression of IL-6, IL-8 and TNF-α target genes in ventral prostate of Wistar rats after treatment with DPPS and DPPE**. All values are expressed as mean ± S.E. of the relative band intensity (R.B.I.) using β-actin as a reference. *Significantly different from normal control rats at P < 0.05. # Significantly different from APH control rats received CMC at P < 0.05.

Induction of APH in rats significantly increased the gene expression of proinflammatory cytokines; IL-6, IL-8 and TNF-α. Saw palmetto treatment exerted a significant reduction in their levels compared to APH-induced rat values. DPPE induced a similar effect to that of saw palmetto in a dose dependent manner. On the other hand, despite DPPS in a dose of 250 mg/kg increased the gene expression IL-6, IL-8 and TNF-α, it induced a dose-dependent reduction of these genes at doses of 500 and 1000 mg/kg (Figure 1).

### Changes in gene expression of TGF-β1, IGF-1 and clusterin

**Figure 2:**
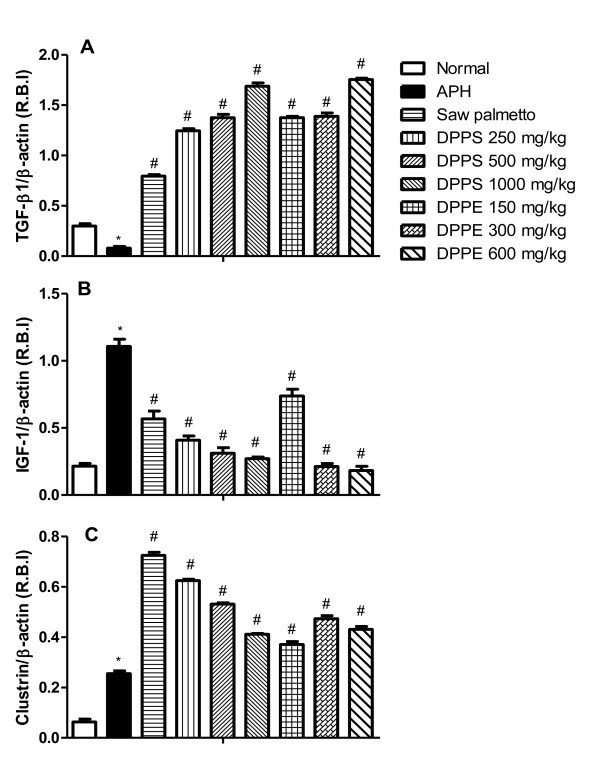
**The expression of TGF-β1, IGF-1 and Clusterin target genes in ventral prostate of Wistar rats after treatment with DPPS and DPPE**. All values are expressed as mean ± S.E. of the relative band intensity (R.B.I.) using β-actin as a reference. *Significantly different from normal rats at P < 0.05. # Significantly different from APH control rats received CMC at P < 0.05.

Induction of prostatic hyperplasia in our model is accompanied with significant increase of the clusterin and IGF-1 expression in ventral lobe of rat prostate, while the TGF-β1 expression was significantly decreased. Saw palmetto treated rats with APH showed significant increase in clustrin and TGF-β1 expression with a decrease in IGF-1 expression. All the treatment groups showed significant increase in clustrin and TGF-β1 expression accompanied with decreasing of IGF-1 expression in a dose dependent fashion (Figure 2).

### Histological changes

**Figure 3:**
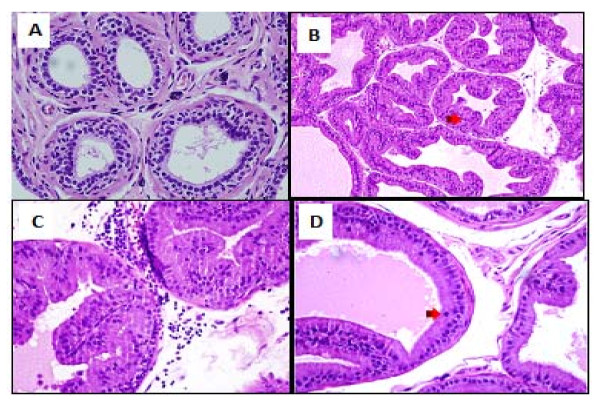
**Prostate of normal rats with round regular acini with an intact basement membranes, the acinar lumina contain eiosinophilic and homogeneous secretions (A), citral and testosterone treated castrated rats with atypical prostatic hyperplasia (APH) showed an irregular acinar growth and distribution, the acini were increased in number, lined by tall columnar epithelium, crowded, irregular in outline and placed back to back with vilous projections (B), the intersititial stroma was scant and showed congested blood vessels with chronic inflammatory cells mainly perivascular lymphocytes (C) hyperplastic acini showing mitosis (D)**.

Ventral prostates from the control normal group showed normal histological pattern with regular acini and average histoscore of 23.3 ± 1.09 units (Table 4). Ventral prostates from the APH-induced rats showed an increased irregular acinar growth and distribution. The acini were placed back to back with vilous projections. The lining epithelium was tall columnar with abundant eosinophilic cytoplasm. Nuclei were round, regular and basally placed in most acini. Epithelial pilling with focal loss of polarity and budding into surrounding stroma was seen in some prostatic sections. The basement membrane was thin and continuous. Isolated mitotic figures were seen in the range of 1-2/section examined. The interstitial stroma was scant and showed congested blood vessels with chronic inflammatory cells mainly lymphocytes and perivascular. These findings were associated with significant increase in the histoscore (Table 4 and Figure 3).

**Table 4:** Histoscore and inflammatory scores in normal rats, atypical prostatic hyperplasia (APH)-induced rats and APH-induced treated treated with date palm pollen suspension (DPPS) and the alcoholic extract (DPPE) (n = 7 rats in each group)

Treatment group	Histoscore	Inflamm-atory score	Number of cases of inflammati-on/tested rats	Percentage inflammation	Percentage inhibition of inflammation
Normal rats	23.30 ± 1.09	0	0	-	-
Rats with APH (negative control)	40.14* ± 1.97	1	7	100*	0
Saw Palmetto (100 mg/kg)	35.28* ± 2.37	1	1	14.28*	85.72^#^
DPPS (250 mg/kg)	36.00* ± 2.21	1	1	14.28*	85.72^#^
DPPS (500 mg/kg)	34.20* ± 2.75	1	1	14.28*	85.72^#^
DPPS (1000 mg/kg)	34.70* ± 1.58	1	0	0	100^#^
DPPE (150 mg/kg)	36.40* ± 2.51	1	1	14.28*	85.72^#^
DPPE (300 mg/kg)	34.20* ± 1.96	1	1	14.28*	85.72^#^
DPPE (600 mg/kg)	34.70* ± 1.97	1	1	14.28*	85.72^#^

*Significantly different from normal control rats at p < 0.05

^# ^Significantly different from APH control rats received CMC at p < 0.05

Score 0: No inflammation

Score 1: Scattered inflammatory cells without nodules

Score 2: No confluent lymphoid aggregates

Score 3: Large areas of confluence

**Figure 4:**
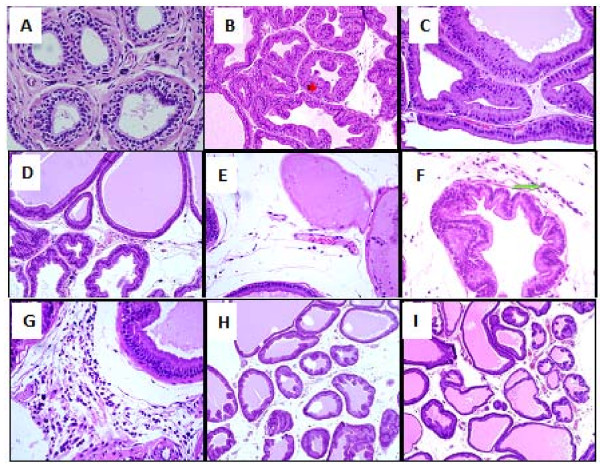
**Normal rats ventral prostate (A), APH in the ventral rat prostate (B), Saw palmetto treated rats prostate showed a persistent hyperplastic acini that were lined by tall columnar epithelium with villous projections and rare chronic inflammatory cells in the stroma (C), DPP suspension (DPPS) showing decreased number of hyperplastic acini in the overall distribution of the lesion (D), hyperplastic acini showing focal loss of lining epithelium (E) few mast cells in the surrounding stroma along with edema (F) and occasional mixed acute and chronic inflammatory cells were seen (G)**. The prostate of the DPPE treated rats, showed a decrease in the number of acini in the overall distribution of the lesion (H) and dilated acini with intraluminal secretions, mitotic figures and chronic inflammatory cells were absent in most cases and surrounding stroma was edematous (I).

These pathological changes and histoscore were ameliorated in animals in the saw palmetto group (Table 4 and Figure 4). However, irregular and back to back acini, with focal pilling of epithelium, were persistent in isolated and multiple foci within the prostatic sections. These acini were lined by tall columnar epithelium and showed round, regular basally placed nuclei. No loss of polarity or budding into surrounding stroma was seen. Isolated mitotic figures in the range of 1-2/section were seen. The interstitial stroma was scant and showed vascular congestion. Rare chronic inflammatory cells were seen.

Similarly, DPPS (250, 500, 1000 mg/kg) and DPPE (150, 300, 600 mg/kg) ameliorated both the pathological features and histoscore of APH (Table 4 and Figure 4). However, persistent hyperplastic changes were seen with an uneven distribution. Most of the affected acini were dilated with intraluminal secretions. Few acini showed focal loss of lining epithelium although the basement membrane was still intact. Mitotic figures were absent in most cases and surrounding stroma showed edema. Occasional mixed acute and chronic inflammatory cells were seen along with few mast cells. Regarding the inflammatory score, treatment with saw palmetto, DPPS and DPPE showed significant inhibition of inflammation nearly in a non-dose dependent manner (Table 4).

### Immunohistochemistry

**Figure 5:**
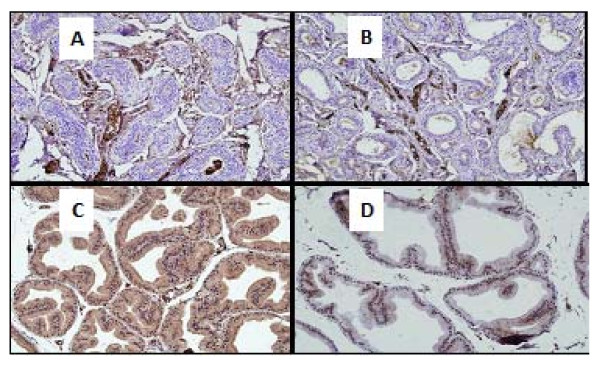
**Immunohistochemical identification and quantification of TGF-βI and IL-6 of: Normal rat prostate TGF-βI score 0 (A), Normal rat prostate IL-6 score 0 (B) APH control rat prostate TGF-βI score 3 (C) and APH control rat prostate IL-6 score 1 (D)**.

Normal control rats showed no expression of all the three immunohistochemical markers. In the prostatic sections from APH-induced rats, TGF-β1 expression score was 3 and showed strong intensity in the secretory epithelial cells of acini with focal strong expression in basal cells as well. IL-6 expression score was 1 and showed weak intensity in the secretory epithelial cells only. No expression was observed in secretory, basal or stromal cells (Figure 5).

In saw palmetto treated APH-induced rats, TGF-β1 expression score was 3 and showed strong intensity in the secretory epithelial cells of acini with focal strong expression in basal cells as well. IL-6 was variable between score 2-3 with a staining intensity between moderate to strong. No expression was observed in secretory, basal or stromal cells.

**Figure 6:**
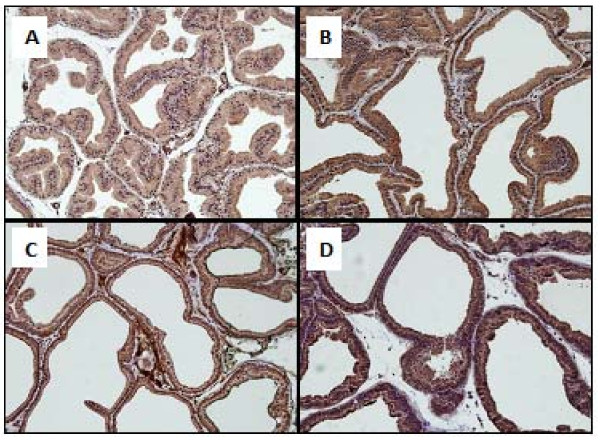
**Immunohistochemical identification and quantification of TGF-βRI, in APH control rat prostate score 2-3 (A), APH rat prostate treated with Saw Palmetto 100 mg kg score 3 (B), APH rat prostate treated with DPPS (1000 mg kg) score 3 (C) and APH rat prostate treated with DPPE (600 mg kg) score 3 (D)**.

**Figure 7:**
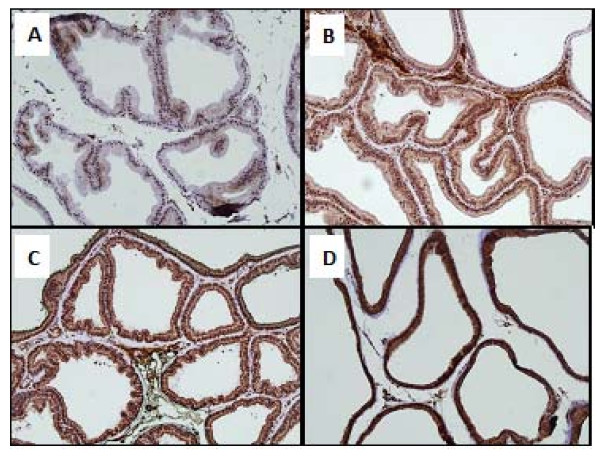
**Immunohistochemical identification and quantification of IL-6 in; APH control rat prostate score 1 (A), APH rat prostate treated with Saw Palmetto (100 mg/kg) score 2-3 (B), APH rat prostate treated with DPPS (1000 mg/kg) score 3 (C) and APH rat prostate treated with DPPE (600 mg/kg) score 3 (D)**.

In DPPS (250 mg/kg) treated rats, TGF-βR1 expression score was 2 and showed moderate intensity in the secretory epithelial cells of acini with focal moderate expression in basal and stromal cells as well. IL-6 expression score was 2 with a strong staining intensity. No expression was observed in secretory, basal or stromal cells (Figures 6 and 7).

In DPPS (500 mg/kg and1000 mg/kg) treated rats, TGF-β1 expression was score 3 and showed strong intensity in the secretory, basal epithelial cells of acini and stromal cells. IL-6 expression score was 3 with a strong staining intensity in secretory and stromal cells. The basal cells also showed focal strong expression of IL-6 with score 3. No expression was observed in secretory, basal or stromal cells. Although the immunoscore enhanced with increase in dose of DPPS from 250 mg/kg to 500 mg/kg, but no statistically significant dose related correlation was observed between a dose of 500 mg/kg and 1000 mg/kg.

In prostatic sections examined from the DPPE (150 mg/kg) treated rats, TGF-β1 expression was variable between score 2-3 and showed moderate to strong intensity in the secretory and basal epithelial cells of acini and stromal cells. IL-6 expression was score 3 with a strong staining intensity in secretory and stromal cells. The basal cells showed focal strong expression with score 3. No expression was observed in secretory, basal or stromal cells

In DPPE (300 mg/kg and 600 mg/kg) treated rats, TGF-β1 expression score was 3 and showed strong intensity in the secretory and basal epithelial cells of acini and stromal cells. IL-6 expression was score 3 with a moderate to strong staining intensity in secretory and stromal cells. The basal cells showed focal strong expression with score 3. No expression was observed in secretory, basal or stromal cells. Although the immunoscore enhanced with increase in dose of DPPE from 150 mg/kg to 300 mg/kg, but no statistically significant dose related correlation was observed between a dose of 300 mg/kg and 600 mg/kg (Figures 6 and 7).

## Discussion

The fact that APH arises always in prostates with concomitant BPH and exhibits several cancer like features, places APH as an intermediate lesion between BPH and the subset of well-differentiated prostatic cancers according to Bostwick et al. [23]. The pathogenesis of prostatic hyperplasia still remains largely unresolved, but a number of partially overlapping and complementary theories have been proposed. In the current study, APH-induced rats revealed prostatic enlargement as a consequence of progressive acinar hyperplasia that confirmed the influence of androgen on prostate growth. These findings are in agreement with that of Abramovici et al. [24]. The development of hyperplasia was associated with enhanced proliferation and suppressed apoptosis of prostatic cells. The APH-induced rats showed an increase in both absolute and relative weights of ventral prostate lobe. This effect was accompanied by histopathological changes confirming the APH which was in agreement with that of Abramovici et al. [24] and Engelstein et al. [17]. It was suggested that APH induced in adult rats by citral may be attributed to its estrogenic-like effect through its binding nonspecifically to estrogen receptors [25] located on prostate epithelial cells of both human [26] and rat [27]. Ho, et al. [27] also found that induction of type 2 estrogen-binding site in rat ventral prostate could be responsible for the proliferative response.

The hyperplastic changes induced in ventral prostate of rats in the current model were accompanied with an increased gene expression of IGF-1, IL-6, IL-8, TNF-α and decreased TGF-β1. TGF-β1 is the only known growth factor that can suppress tissue proliferation and induce cell apoptosis [28]. Circulating testosterone acts locally in the prostate via the production of growth factors such as IGF and TGF families that act in an autocrine or paracrine manner to influence prostate cell growth, survival, or apoptosis [29]. There are essentially two major classes of growth factors that are complimentary due to their effects on cell growth, namely growth stimulatory or mitogenic factors such as IGFs and growth inhibitory factors, including TGF-β1. The prostate enlargement and the increased net weight of the ventral lobe may be partly due to, the increased IGF-1 and decreased TGF-β1expression. This modulation in expression of these cytokines may play a crucial role in prostate cell proliferation and apoptosis. These finding are in agreement with that of Wu et al. [30]. IGF-1, a mitogen, interacts with IGF-1 receptor stimulating cell proliferation [31] and inducing proliferative prostatic diseases [32].

Inflammation may play a role in prostatic hyperplasia [33]. About 40% of patients with BPH and APH exhibit chronic inflammatory features including infiltration by activated T cells, mast cells and macrophages [34]. Results of the present study showed that the enlargement of the ventral prostate of rats may be in part due to inflammation and over-expression of the pro-inflammatory cytokines including IL-6, IL-8 and TNF-α. It was reported that the infiltrated cells are capable of secreting growth factors and proinflammatory cytokines such as IL-6, IL-8, TGF-β1 and IFN-α [33]. IL-6 is also secreted by both normal and prostatic epithelial cells and acts as a growth factor for normal prostatic epithelial cells [35]. IL-8 has chemotactic properties, attracting neutrophils and mononuclear cells into the sites of inflammation. It is a primary inflammatory cytokine, secreted by monocytes, mitogen-stimulated T lymphocytes, and many other prostatic cells [36]. IL-8 mRNA expression was upregulated as much as 5-fold in peripheral blood lymphocytes of patients with prostatic carcinoma and in prostate tissues with hyperplasia and can act as a paracrine inducer of some potent growth factors for prostatic stromal cells [37]. TNF-α, a pro-inflammatory cytokine, may induce inflammation by induction of COX-2, superoxide and hydrogen peroxide in human and rat mesangial cells [38]. TGF-β, an inflammatory cytokine, has been shown to regulate stromal proliferation and differentiation in BPH, and it is a key factor for androgen control of prostatic growth. Recently, Descazeaud et al [39] investigated the TGF-β receptor II protein (TGFBRII) expression in 231 BPH patients using large-scale tissue microarrays analysis. They observed a significant association between TGFBRII stromal staining and prostatic volume.

Results of the present study showed that administration of DPPS and DPPE has ameliorated the histopathological changes and pro-inflammatory cytokine expression in APH-induced rats in a dose dependent manner as well as the inflammatory score in a non-dose dependent manner. These findings are in agreement with that of Ito et al. [40]. They reported that cernilton, the water soluble fraction of pollens extract of different plants, produced histological evidence of epithelial cell atrophy and significant reduction in the size of ventral and dorsal lobes of prostate in mature Wistar rats. These effects may be due to the anti-inflammatory activity of the DPP. In the context of chronic inflammation and expression of proinflammatory cytokines, IL-6 is one of the major physiologic mediators of acute phase reaction that influence immune responses and inflammatory reactions [36].

Pollen extract contains five different phytosterols and a biological active peptide that can influence the intracellular metabolism in biological systems [13]. Pollen extract showed a decrease in the leucocyte number in the prostatic expressate in 59% of all cases [13]. The water-soluble fraction attenuated the inflammatory response in experimental animals [41]. The in vitro experiments suggested that GBX (an acetone-soluble cernitin pollen extract) could be either a smooth muscle relaxant or a potent cyclo-oxygenase and lipoxygenase inhibitor [14]. The selectivity of pollen extract for the prostate was also supported by the work carried out by Ito et al. [40]. The homogenous effect of pollen-extract on the bladder outflow obstruction, showed higher values of urine volume voided and reduced flow time in both prostatitis and BPH [13]. The anti-inflammatory and decongestive effects of pollen-extract, give the evidence for the therapeutic benefit in patients with non-bacterial prostatitis or prostodynia [13,42]. The significant decrease in the A-P diameter of the prostate in patients treated with cernilton suggests that the prostate size was reduced. Nakase et al. [43] reported that T-60 (a water-soluble cernitin pollen extract) and GBX inhibited the contraction of muscle induced by noradrenaline, with evidence for competitive antagonism of noradrenalin at the site of adrenergic receptors. The subjective improvement in symptoms of nocturia and bladder emptying could be due to the effect of pollen extract on the rich adrenergic innervation of the bladder neck and prostate [44]. Similar findings were recorded in the present study in APH-induced rats treated with saw palmetto. Saw plametto was reported to inhibit growth factors-induced cell proliferation [45]. It has been reported that the liposterolic extract of Saw palmetto has antiandrogenic effects, [46] and inhibits the type 1 and type 2 isoenzymes of 5-alpha-reductase [47].

The reduction in the number of acini and the absence of mitotic figures in the surrounding stroma during treatment with both DPPS and DPPE may be due to increased tissue apoptosis and decreased proliferation. Results of the present study showed that DPP increased the expression of TGF-βI and clusterin while decreased the expression of IGF-1 in rat ventral lobe prostate. Overexpression of TGF-β1 and clusterin showed the increased rate of apoptosis of acini on the ventral prostate.

## Conclusion

The findings of the present study clearly suggest that suspension or extract of DPP has ameliorating effect on the prostatic hypertrophy and inflammation. DDP treatment reduced the number of prostatic acini in APH rat model and decreased the production of pro-inflammatory cytokines. Prostatic tissues proliferation was decreased and apoptosis was increased during the treatment with DPP suspension and extract. The watery edema found during DDP treatment was responsible for the increased volume and hence the weight of the ventral lobe. This may explain the non-significant change of ventral prostate weight after treatment despite of the anti-inflammatory, antiproliferative and apoptotic effects. Further studies may be required for longer time treatment to study the effects of DPP on the absolute and relative weight of ventral lobe as well as the total net weight of the prostate in this model of APH in rats.

## Competing interests

The authors declare that they have no competing interests.

## Authors' contributions

SG and HM designed the study. AE, OA and SG executed the experiments in the manuscripts. EA was responsible for collection and extraction of date pollen. SM and JA were involved in histopathological and immunohistochemical study as well as data analysis and manuscript preparation. All authors read and approved the final manuscript.
